# Placental growth fActor Repeat sampling for Reduction of adverse perinatal Outcomes in women with suspecTed pre-eclampsia: study protocol for a randomised controlled trial (PARROT-2)

**DOI:** 10.1186/s13063-022-06652-8

**Published:** 2022-09-02

**Authors:** Alice Hurrell, Jenie Sparkes, Kate Duhig, Paul T. Seed, Jenny Myers, Cheryl Battersby, Katherine Clark, Marcus Green, Rachael M. Hunter, Andrew H. Shennan, Lucy C. Chappell, Louise Webster

**Affiliations:** 1 Department of Women and Children’s Health, School of Life Course Sciences, King’s College London, Westminster Bridge Road, London, SE1 7EH UK; 2 Maternal & Fetal Health Research Centre, Division of Developmental Biology and Medicine, School of Medical Sciences, Faculty of Biology, Medicine and Health, University of Manchester, Manchester Academic Health Science Centre, Manchester, M13 9PL UK; 3 Neonatal Medicine, School of Public Health, Faculty of Medicine, Imperial College London Chelsea and Westminster Hospital Campus, 369 Fulham Road, London, SW10 9NH UK; 4 Action on Pre-eclampsia, Evesham, UK; 5 Research Department of Primary Care and Population Health, University College London, London, UK

**Keywords:** Pre-eclampsia, Angiogenic biomarkers, Placental growth factor, Soluble fms-like tyrosine kinase 1

## Abstract

**Background:**

Pre-eclampsia is a complex pregnancy disorder, characterised by new or worsening hypertension associated with multi-organ dysfunction. Adverse outcomes include eclampsia, liver rupture, stroke, pulmonary oedema, and acute kidney injury in the mother, and stillbirth, foetal growth restriction, and iatrogenic preterm delivery for the foetus. Angiogenic biomarkers, including placental growth factor (PlGF) and soluble fms-like tyrosine kinase 1 (sFlt-1), have been identified as valuable biomarkers for preterm pre-eclampsia, accelerating diagnosis and reducing maternal adverse outcomes by risk stratification, with enhanced surveillance for high-risk women. PlGF-based testing for suspected preterm pre-eclampsia has been incorporated into national guidance. The role of repeat PlGF-based testing and its effect on maternal and perinatal adverse outcomes have yet to be evaluated.

**Methods:**

The PARROT-2 trial is a multi-centre randomised controlled trial of repeat revealed PlGF-based testing compared to repeat concealed testing, in women presenting with suspected pre-eclampsia between 22^+0^ and 35^+6^ weeks’ gestation. The primary objective is to establish whether repeat PlGF-based testing decreases a composite of perinatal severe adverse outcomes (stillbirth, early neonatal death, or neonatal unit admission). All women prior to enrolment in the trial will have an initial revealed PlGF-based test. Repeat PlGF-based tests will be performed weekly or two-weekly, depending on the initial PlGF-based test result, with results randomised to revealed or concealed.

**Discussion:**

National guidance recommends that all women presenting with suspected preterm pre-eclampsia should have a single PlGF-based test when disease is first suspected, to help rule out pre-eclampsia. Clinical and cost-effectiveness of repeat PlGF-based testing has yet to be investigated. This trial aims to address whether repeat PlGF-based testing reduces severe maternal and perinatal adverse outcomes and whether repeat testing is cost-effective.

**Trial registration:**

ISRCTN 85912420. Registered on 25 November 2019

**Supplementary Information:**

The online version contains supplementary material available at 10.1186/s13063-022-06652-8.

## Background 

Hypertension affects 10% of pregnant women, with pre-eclampsia affecting 2.8% of singleton pregnancies [1]. Forty per cent of all pre-eclampsia occurs preterm [2] and 12% before 34 weeks’ gestation [3], and preterm pre-eclampsia is more frequently associated with adverse outcomes [4, 5]. Maternal complications include eclampsia, liver rupture, stroke, pulmonary oedema, and acute kidney injury, and foetal/neonatal complications include stillbirth, foetal growth restriction, and iatrogenic preterm delivery [6, 7]. Pre-eclampsia has a variable clinical presentation and women may be asymptomatic even with severe disease. Symptoms may progress over weeks and do not correlate well with diagnosis or clinical outcomes. Conversely, hypertension and proteinuria can occur without progression to pre-eclampsia. Pre-eclampsia also doubles a woman’s lifelong risk of cardiovascular disease [8].

Placental growth factor (PlGF) is an angiogenic protein synthesised in the placenta. The concentration of PlGF in maternal blood rises with advancing gestation to peak at 30 weeks and then falls slightly towards term in uncomplicated pregnancies [9]. In pregnancies affected by pre-eclampsia, PlGF concentration is abnormally low, and this predates the onset of clinical pre-eclampsia. Conversely, PlGF > 5th centile (≥100pg/ml) is a good rule-out test for delivery due to pre-eclampsia for the next 2 weeks, with a high sensitivity and negative predictive value [10]. Soluble fms-like tyrosine kinase 1 (sFlt-1) is a circulating anti-angiogenic protein which adheres to the receptor-binding domains of PlGF and vascular endothelial growth factor (VEGF). sFlt-1 concentrations increase towards term in healthy pregnancies but are prematurely elevated in the serum of women with pre-eclampsia [9]. Abnormalities in angiogenic factors may predate the clinical syndrome of pre-eclampsia by up to 10 weeks [11].

In 2016, the National Institute for Health and Care Excellence published diagnostic guidance recommending PlGF-based testing to help rule out pre-eclampsia, in women presenting with suspected pre-eclampsia after 20 weeks and before 37 weeks’ gestation [12]. They concluded that there was adequate evidence to recommend two of the commercially available PlGF-based tests for clinical use — the Quidel PlGF test and the Roche sFlt-1/PlGF ratio. This recommendation was based on evidence from the PELICAN study, demonstrating that PlGF >5th centile (≥100 pg/ml) rules out pre-eclampsia necessitating delivery within 14 days, with a negative predictive value of 0.98 (95% confidence interval (CI) 0.93–0.995), and the PROGNOSIS study, demonstrating that sFlt-1/PlGF ≤38 rules out pre-eclampsia necessitating delivery within 1 week, with a negative predictive value of 99.3% (95% CI 97.9–99.9) [10, 13–15].

There remains a need to investigate the use of repeat PlGF-based testing, to evaluate the potential impact on maternal and perinatal complications, including stillbirth, neonatal death, and neonatal unit admission. This is particularly important in women in whom a clear risk trajectory or diagnosis is not reached at the initial clinical presentation, but in whom there remains ongoing suspicion of disease. A case series study found that repeat PlGF testing has high diagnostic accuracy, with high sensitivity (90.7%, 95% CI 85.2–95.9%) and negative predictive value (92.2%, 95% CI 85.3–96.6%) [16]. Another study demonstrated, compared to women who did not develop pre-eclampsia, those who did had significantly larger median increases in sFlt-1/PlGF ratios at 2 and 3 weeks after the initial test (*p* < 0.001) [17]. A retrospective study of women with chronic hypertension found that longitudinal changes in sFlt-1/PlGF had a higher area under the curve than the last measurement alone (area under the curve 0.95, 95% CI 0.92–0.99 vs 0.87, 95% CI 0.79–0.95, *p* = 0.02) [18]. However, before repeat testing is recommended, it needs to be established whether it is clinically and cost-effective, and what added benefit repeat PlGF-based testing offers over the initial PlGF-based test result. This was given as an explicit research recommendation in the National Institute for Health and Care Excellence diagnostic guideline [12].

## Methods/design

The aim of this trial is to establish whether repeat PlGF-based testing (using either the Quidel PlGF test or the Roche sFlt-1/PlGF ratio) reduces adverse pregnancy outcomes compared to usual care (including an initial pre-enrolment PlGF-based test), in women presenting with suspected preterm pre-eclampsia.

### Primary objective

The primary objective of the study is to establish whether repeat PlGF-based testing decreases a composite of perinatal severe adverse outcomes, in women who have already had an initial PlGF-based test.

### Secondary objectives

The secondary objectives of the study are to determine if repeat PlGF-based testing reduces secondary perinatal and maternal adverse outcomes and to assess the health resource use associated with repeat PlGF-based testing in a budget impact analysis. The study also aims to establish the diagnostic test accuracy of ‘low’ (PlGF <100 pg/ml) or ‘very low’ (PlGF <12 pg/ml) or ‘high’ sFlt-1/PlGF ratio (>38) repeat PlGF-based tests in predicting pre-eclampsia requiring delivery in 14 days.

### Trial design

The PARROT-2 trial is a pragmatic, multi-centre, randomised controlled trial of repeat revealed PlGF-based testing compared with repeat concealed testing, in women presenting with suspected preterm pre-eclampsia between 22^+0^ and 35^+6^ weeks of gestation inclusive. Women will have received initial PlGF-based testing at presentation within usual clinical care prior to enrolment (as recommended by the National Institute for Health and Care Excellence [19]). Women will be randomised at an individual level and the allocation ratio of intervention (repeat revealed PlGF-based testing) to control (repeat concealed PlGF-based testing) will be 1:1. The trial will be conducted in approximately 20 to 30 consultant-led maternity units across England, Scotland, and Wales.

Women who do not wish to participate in the randomised trial after an initial revealed PlGF-based test will be invited to participate in an observational arm of the study where maternal and neonatal outcome data will be used to assess the generalisability of the main trial findings.

### Selection and withdrawal of participants

Women can self-refer or be referred by a healthcare professional to maternity triage units or other antenatal care settings, for assessment of suspected pre-eclampsia. Those meeting the inclusion criteria will be approached to consider participation.

### Inclusion criteria

Women will be considered eligible for inclusion into the trial if they fit the following criteria at the time of the initial PlGF-based test:

- Clinical suspicion of pre-eclampsia
- Pregnancy of between 22^+0^ and 35^+6^ weeks’ gestation inclusive
- Singleton pregnancy
- Viable foetus
- Women aged 18 years or more at the time of presentation
- Able to give written informed consent

### Exclusion criteria

Women will be excluded from the trial if they have a confirmed diagnosis of preterm pre-eclampsia at the time of the initial PlGF-based test.

### Study period

A woman’s participation in the study may be from 22 weeks’ gestation until the primary discharge of the woman and her baby after birth. Women may participate in the study more than once if they have a second pregnancy whilst the study is still running, and they are eligible to participate. Outcome collection will end 42 days after the final recruited participant has given birth.

### Withdrawal of participants

Women will be free to withdraw from taking part in the trial at any time and without giving a reason. Withdrawal from the study will not affect any aspect of ongoing clinical care. Permission will be sought to ascertain and record subsequent perinatal and maternal outcome data.

If a participant, who has given informed consent, loses the capacity to consent during the trial, the participant would be withdrawn from the study. Identifiable data or samples already collected with consent would be retained and used in the study. No further data or samples would be collected, or any other research procedures carried out on or in relation to the participant.

### Assessment of outcomes

Outcome data will be recorded on the Web-based database after a review of case notes by trained researchers. Each outcome considered a case of the primary outcome will have the case notes reviewed by the site principal investigator or delegate and by the central trial team to ensure the case definition is met.

### Primary outcomes

The primary outcome is a composite of stillbirth, or early neonatal death, or neonatal unit admission.

### Secondary outcomes

Tested perinatal outcomes:

- Stillbirth
- Early neonatal death (within 7 days of delivery)
- Neonatal unit admission (physical separation of the baby from the mother)
- Gestational age at delivery
- Birthweight centile <10th (using Intergrowth-21st standards)
- Survival to discharge without severe morbidity [20]: defined as survival to neonatal discharge without any of the following: bronchopulmonary dysplasia, retinopathy of prematurity, severe necrotising enterocolitis, brain injury, late-onset sepsis

Additional descriptive perinatal outcomes (as captured by routine clinical descriptors and listed in the clinical discharge summary):

- Late neonatal death (within 28 days of delivery)
- Birthweight <3rd centile (using Intergrowth-21st standards)
- Severe necrotising enterocolitis (using the UK Neonatal Collaborative definition, confirmed at surgery, histology, post-mortem, or causing death) [21]
- Sepsis (defined as one or more episodes of a positive blood or cerebrospinal fluid culture with either a pure or mixed growth of a known pathogenic organism, subdivided into early-onset sepsis and late-onset sepsis) [22]
- Brain injury: seizures, intracranial haemorrhage, perinatal stroke, moderate/severe hypoxic-ischaemic encephalopathy or hypoxic-ischaemic encephalopathy requiring therapeutic hypothermia, cystic periventricular leukomalacia, left or right, grade 3, or higher intraventricular haemorrhage
- Seizures: any clinical/confirmed by electroencephalogram/treated medically
- Retinopathy of prematurity (defined as requiring cryotherapy, laser therapy, or injection of anti-vascular endothelial growth factor therapy in one or both eyes) and maximum stage of retinopathy of prematurity in either eye (stages 1–5)
- Chronic lung disease or bronchopulmonary dysplasia (defined as supplemental oxygen requirement and/or receiving respiratory support at 36 weeks postmenstrual age)
- Umbilical arterial pH at birth (where measured)

Maternal tested secondary outcomes (between enrolment and delivery):

- Proportion of women diagnosed with pre-eclampsia (defined by International Society for Study of Hypertension in Pregnancy) [7]
- Severe adverse maternal outcome composite (defined by fullPIERS consensus) [23]
- Systolic blood pressure ≥160 mmHg (with or without medication)
- Delivery mode (vaginal, assisted vaginal, caesarean section)

Concealed first repeat PlGF-based test performance (with comparison against currently utilised tests) for clinically indicated delivery for diagnosed pre-eclampsia within 14 days will be reported.

Additional descriptive maternal outcomes:

- Components of the fullPIERS composite (as coded in routine clinical documentation and verified by two members of the central research team) [23]:
  - ◦ Maternal death
  - ◦ Eclampsia
  - ◦ Glasgow coma score <13
  - ◦ Stroke or reversible ischaemic neurological deficit
  - ◦ Transient ischaemic attack
  - ◦ Cortical blindness or retinal detachment
  - ◦ Posterior reversible encephalopathy
  - ◦ Positive inotropic support
  - ◦ Severe uncontrolled hypertension, with parenteral infusion of third-line antihypertensive required
  - ◦ Myocardial infarction/ischaemia
  - ◦ Blood oxygen saturation <90%
  - ◦ Requirement of ≥50% FiO2 for >1 h
  - ◦ Intubation required (other than for caesarean section)
  - ◦ Pulmonary oedema
  - ◦ Transfusion of blood products required
  - ◦ Platelet count <50 × 10^9^ platelets/L
  - ◦ Hepatic dysfunction (INR >1.2 in the absence of disseminated intravascular coagulopathy or treatment of warfarin)
  - ◦ Hepatic haematoma or rupture
  - ◦ Severe acute kidney injury (creatinine >150 μmol/L; no pre-existing renal disease or creatinine >200 μmol/L; pre-existing renal disease)
  - ◦ Dialysis required
  - ◦ Placental abruption
- Abnormal foetal ultrasound features post-enrolment such as estimated foetal weight <10th centile, oligohydramnios, or absent or reversed umbilical artery Doppler end-diastolic flow
- Labour onset (spontaneous, induced, or pre-labour caesarean section)
- Indications for delivery
- Postpartum haemorrhage

Health resource use outcomes:

- Maternal:
  - ◦ Antenatal outpatient attendances
  - ◦ Ultrasound scans
  - ◦ Inpatient days
  - ◦ Intensive care unit days
- Perinatal:
  - ◦ Intensive care unit days
  - ◦ High dependency unit days
  - ◦ Special care unit days

The cost of repeat PlGF-based testing will be included for those in the intervention group.

### Trial procedures

**Fig. 1:**
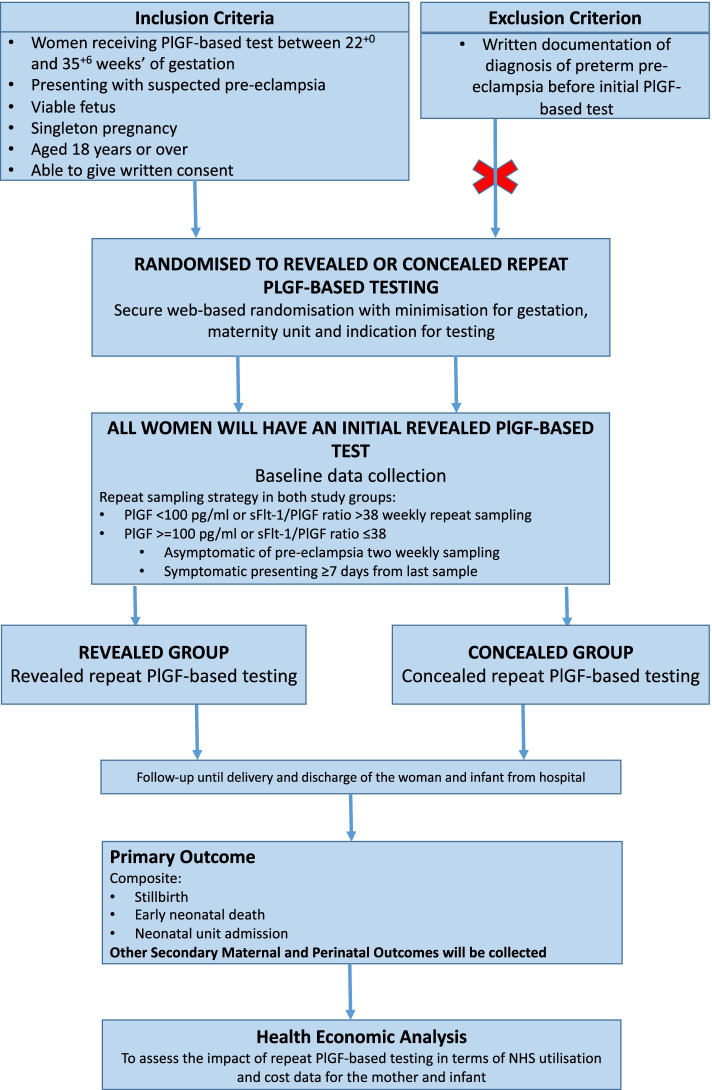
Trial procedures

The trial procedures are shown in Fig. 1, and trial assessments and interventions are shown in Table 1.

**Table 1:** Schedule of participant enrolment, interventions, and assessment in the trial

	Screening^a^	Trial entry	Trial period	At hospital discharge to home
Confirmation of eligibility	**✓**			
Consent		**✓**		
Demographic/clinical data collection		**✓**		
Subsequent blood sample(s) for PlGF-based test (revealed or concealed as per randomised allocation)			**✓**	
SAEs			**✓**	**✓**
Post-delivery outcome data collection				**✓**

^a^Screening to be recorded of all women considered possibly eligible for the study

### Informed consent

Members of the research team will provide a full verbal explanation and written description of the trial to women who meet the inclusion criteria (as in the participant information leaflet; Additional file 1). Women will be given sufficient time to consider the information and to decide whether they wish to participate. Women who agree to participate will give written informed consent (as in the informed consent form; Additional file 2). Where the English language is limited, an adult interpreter can be used to translate the study materials and ensure the woman understands all that is involved with participation in the trial prior to signing consent.

After written informed consent has been obtained by a member of the research team with delegated authority, baseline details will be entered onto the online database and randomisation performed, with direct communication of the allocation to the woman. At all stages, it will be made clear to the woman that she is free to withdraw from the trial at any time without the need to provide any reason or explanation, and this will have no impact on any aspect of ongoing care. Clinical management should be in accordance with the National Institute for Health and Care Excellence guidelines for the management of hypertension in pregnancy and the management algorithm incorporating PlGF results, if available according to the randomisation (Additional file 3, management algorithm) [19]. All options should be discussed with the pregnant woman and her needs and preferences taken into account.

### Pre-enrolment

All women will have an initial revealed PlGF-based test, in line with current guidance [19], allowing the treating clinician to formulate an individualised clinical management plan using guidance from the test result integrated with the National Institute for Health and Care Excellence Hypertension in Pregnancy Guideline.

### Intervention group (repeat revealed PlGF-based testing)

The Quidel PlGF test is a single-use, fluorescence immunoassay device, which is used with the CE-marked Triage MeterPro point-of-care analyser. Blood must be centrifuged, and plasma extracted before testing. The test takes approximately 40 min to analyse a result (including centrifugation). It detects PlGF-1 and quantifies concentration in the range of 12 to 3000 pg/ml.

The Roche Elecsys immunoassay sFlt-1/PlGF ratio measures the amounts of PlGF relative to sFlt-1 in serum. The ratio combines the results from 2 CE-marked sandwich electrochemiluminescence immunoassays (Elecsys PlGF and Elecsys sFlt-1 assays). The turnaround time is 18 min on the analyser, but in reality, it takes longer due to laboratory processing. The Elecsys sFlt-1 assay has a lower limit of detection of 10pg/ml, a range of 10–85,000pg/ml. The Elecsys PlGF assay has a lower limit of detection of 3pg/ml and a range of 3 to 10,000pg/ml.

PlGF-based immunoassays are NICE-approved diagnostic tests for the initial assessment of suspected pre-eclampsia [12, 19]. All regulatory approvals are in place. PlGF and sFlt-1 are stable markers, and the collection of blood samples is straightforward, requiring no additional processes beyond centrifugation (as used in routine clinical blood sampling). Coefficients of variation have been established for the assays and are acceptable for use in clinical practice.

The results of the repeat PlGF-based test will be revealed to the healthcare professionals and the women in the intervention arm and used in addition to the other clinical features to inform the ongoing management plan integrated with the National Institute for Health and Care Excellence Hypertension in Pregnancy Guideline [19]. Clinical staff will be trained in the interpretation of PlGF-based test results and provided with a management algorithm to be integrated into the participant’s clinical care (see Additional file 3).

### Control group (repeat concealed PlGF-based testing)

All repeat concealed tests will be spun, and plasma/serum extracted and stored at −80°C. The samples will be batch processed at the co-ordinating centre or collaborating sites, once the women have delivered and the results will remain concealed to the research team until the trial has completed all participant follow-up.

### Sample scheduling

For the trial, the women will be asked to provide one extra tube of blood (as far as possible at the same time as clinical blood samples) up to four times during the rest of their pregnancy according to the schedule below. It is recognised that some women will only provide one sample; from previous studies, women may provide a variable number of samples depending on their interval from the first test to delivery [10].

For both the revealed repeat testing and concealed repeat testing groups, the repeat sampling strategy will be based on the first PlGF test result as follows:

- If PlGF <100 pg/ml or sFlt-1/PlGF ratio >38, i.e. higher risk, sampling will be weekly (± 2 days) whilst attending for clinical review.
- If PlGF ≥100 pg/ml or sFlt-1/PlGF ratio ≤38 (lower risk) and asymptomatic of pre-eclampsia, sampling will be every 2 weeks (± 7 days) whilst attending for routine antenatal checks. If a woman presents ≥7 days from the last sample and is symptomatic, an additional sample can be taken and reported.

### Sample size

The sample size was calculated using data from the PELICAN study and PARROT-1 trial combined [10, 24], demonstrating that 25.7% had the primary outcome (stillbirth, early neonatal death, or neonatal unit admission). A sample size of 1208 women (604 participants per group) would have 90% power, at the 5% significance level, to detect an overall reduction of 30% (to 18.0%) in the composite primary outcome score. Although loss to follow-up in the PARROT-1 trial was three of 1023 women, we will allow for up to 5% loss to follow-up and plan to recruit 1268 women in this trial. If 3% of women are lost to follow-up, a sample size of 1244 would be sufficient.

This analysis will primarily assess a PlGF-based testing strategy, using one of the two tests approved by the National Institute for Health and Care Excellence (Roche and Quidel). If we recruit 650 women into the trial using each of the Roche PlGF-based test (sflt-1/PlGF), or the Quidel test, then if analysed as its own group, this would give 90% power to detect a reduction in the composite events from 25.7 to 15.4% (40% relative risk reduction) or 80% power to detect a reduction in the composite events from 25.7 to 16.7% (35% relative risk reduction).

Sample size table

**Table:** 

**Baseline event rate — 25.7%**	**90% power**	**80% power**
25% RRR to 19.3%	1786	1336
30% RRR to 18.0%	1208	902
35% RRR to 16.7%	864	646
40% RRR to 15.4%	644	482

### Impact of COVID-19 and mitigation

The PARROT-2 trial has been conducted during the COVID-19 pandemic, with repeat disruption at recruiting sites and adjustments to the delivery of maternity care. The trial has been formally suspended at some participating sites during peaks of the pandemic. Recruitment at all sites has been impacted by the re-deployment of research staff to clinical roles, staff shortages due to isolation/sickness, and prioritisation of COVID-19 research. Follow-up and repeat testing have been affected by adjustments to antenatal care, with an increase in virtual monitoring of women.

Whilst we initially aimed to recruit 1208 women to each of the two PlGF-based testing strategies (the Quidel PlGF test and Roche sFlt-1/PlGF ratio), the effect of the COVID-19 pandemic has meant we will primarily assess PlGF-based repeat testing as a whole, with further analysis as outlined in the Statistical Analysis Plan.

### Randomisation

Randomisation will be managed via MedSciNet, a secure Web-based randomisation facility. The allocation ratio of intervention (repeat revealed PlGF-based testing) to control (repeat concealed PlGF-based testing) will be 1:1. Participants will be randomised as soon as they have signed consent to participate in the study. A minimisation algorithm will be used to ensure balance between the groups with respect to the maternity unit, gestational age at randomisation (22^+0^ to 27^+6^, 28^+0^ to 31^+6^, ≥32^+0^ weeks’ gestation), and primary indication for testing (hypertension, others).

The MedSciNet web-based randomisation, using a minimisation algorithm as described above, will ensure that the mechanism for deriving the allocation is not shared with researchers, clinicians, and participants. The randomisation algorithm will be checked prior to trial initiation, with data from the PARROT-1 trial.

### Masking

Due to the study design, it is not possible to mask allocation from the clinical researchers or the women who are recruited to the trial. However, the study team will take steps to ensure that those participants assigned to the concealed repeat sampling arm of the trial do not have any repeat revealed tests. Data analysts will be blinded to the allocation.

### Data collection

Outcome data will be collected using bespoke electronic case report forms and entered directly onto the study’s electronic database. It is expected that data on all outcomes should be completed by 6 weeks after delivery. Loss to follow-up was <1% in the recent PARROT-1 study (11 centres) and every effort will be made to follow up women who deliver out of the study centre. Outcome data will be collected by the centre’s research team, with the principal investigator (PI) or a nominated deputy providing a second sign-off for all primary outcomes.

The PARROT-2 trial management team will monitor recruitment against targets and perform a number of validation checks to verify validity and completeness. A minimum of 10% of participants from each site will have their outcomes independently validated by the central trial team. Training in the trial protocol and procedures will be delivered either at the site or centrally (before recruitment begins), to ensure staff are confident and competent to recruit women to the trial and collect outcome data.

### Assessment of safety

A Data Monitoring Committee (DMC) will be established to ensure the wellbeing of study participants. The DMC will periodically review study progress and outcomes as well as reports of unexpected and serious reportable SAEs as defined below. The DMC will, if appropriate, make recommendations to the Trial Steering Committee (TSC) regarding the continuance of the study or modification of the study protocol.

#### Adverse events

An adverse event is any untoward medical occurrence in a participant, which does not necessarily have a causal relationship with this intervention. Due to the high incidence of adverse events routinely expected in this patient population (e.g. abnormal laboratory findings, new symptoms, etc.), only those adverse events identified as serious will be recorded for the trial.

#### Serious Adverse Events (SAEs)

A serious adverse event is any untoward medical occurrence that:

- Results in death
- Is life-threatening
- Requires inpatient hospitalisation or prolongation of existing hospitalisation
- Results in persistent or significant disability/incapacity
- Consists of a congenital anomaly/birth defect

#### Expected SAEs

Expected SAEs are those events which are expected in the patient population or as a result of the routine care/treatment of a patient. These have been separated into expected SAEs that are reportable and those that are not.

The following events are expected in women with pre-eclampsia and their infants and will be recorded comprehensively on the maternal/infant outcomes section of the clinical record form. They do not require reporting as SAEs.

#### Expected maternal SAEs

- Expected complications of pre-eclampsia (including but not limited to those listed in the fullPIERS composite [23])
- Admission to hospital for pregnancy-related monitoring, or monitoring for other medical or psychiatric condition in pregnancy, or delivery or other complication related to pregnancy
- Admission to a high dependency unit or intensive care unit for an expected complication

#### Expected infant SAEs

- Neonatal unit admission and associated morbidity
- Transitional care (for example hypoglycaemia and hypothermia)
- Congenital anomaly

Although it is known that maternal death, eclamptic fits, and stroke can occur in a woman with pre-eclampsia, they should still be reported as an SAE. Additionally, although it is known that stillbirth and neonatal death can occur in infants born to women with pre-eclampsia, they should still be reported as an SAE.

#### Expected reportable SAEs

- Maternal death
- Maternal stroke
- Maternal eclamptic seizure
- Maternal cardiac arrest
- Stillbirth or neonatal death

#### Unexpected SAEs

An unexpected serious adverse event is one which is not anticipated and is not known to be related to the condition being studied or the intervention being assessed. Unexpected SAEs will be collected and the relatedness of the SAE to the intervention will be assessed.

#### Safety reporting procedures

All SAEs will be recorded from randomisation to the primary postnatal discharge from the hospital of the mother and baby. Unexpected related SAEs or expected reportable SAEs listed above for both the mother and the baby will be recorded and reported by the chief investigators (CI) to the DMC.

A SAE occurring to a participant will be reported to the Research Ethics Committee (REC) that gave a favourable opinion of the study where in the opinion of the chief investigators the event was ‘related’ (resulted from the administration of any of the research procedures) and ‘unexpected’ in relation to those procedures. Reports of related and unexpected SAEs will be submitted within 15 working days of the chief investigators becoming aware of the event, using the HRA report of serious adverse event form.

All reported SAEs will be reviewed by the DMC at regular intervals throughout the study. The chief investigators will inform all principal investigators of relevant information that could adversely affect the safety of participants.

### Statistical analysis

The main analysis will follow the intention-to-treat principle, with all randomised participants analysed in their original groups. All analyses will be carried out using a two-sided type 1 error rate of 0.05. The binary composite of stillbirth, early neonatal death, or neonatal unit admission will be analysed using binomial regression with a log link, adjusted for the minimisation variables (maternity unit, gestational age at randomisation (22^+0^ to 27^+6^, 28^+0^ to 31^+6^, >32^+0^ weeks’ gestation) and primary indication for testing (hypertension, other). Results will be presented as a risk ratio with 95% confidence intervals. In general, logistic regression and odds ratios will only be used if the binomial model fails to converge.

The tested secondary perinatal and maternal outcomes will be analysed using log binomial regression models and results will be presented as adjusted risk ratios with 95% confidence intervals. Continuous outcomes will be analysed using linear regression with log transformations as necessary. Additional perinatal and maternal outcomes will be reported using descriptive statistics alone. A full statistical analysis plan can be found in Additional file 4.

### Test performance analysis

Within the concealed group, the diagnostic accuracy of the first repeat sample will be assessed for pre-eclampsia requiring delivery in 14 days (the commonly used outcome in previous diagnostic test accuracy studies). Sensitivity; specificity; positive and negative predictive value; positive and negative likelihood ratios, using cut points of 12 and 100 pg/mL for the Quidel PlGF test and 38 for the Roche sFlt-1/PlGF ratio; and area under the receiver operating characteristic curve will be reported with 95% confidence intervals. Exploratory analyses will be undertaken looking at other cut-offs.

### Economic evaluation

A health economic analysis will be undertaken, to evaluate the resource implications of repeat PlGF-based sampling as part of a management algorithm, compared with current practice, similar to that done by our group previously for the PELICAN study [10] and PARROT-1 trial [24]. A full health economics analysis plan can be found in Additional file 5.

Data on mother and infant, antenatal and post-natal acute hospital care (hospital attendances including outpatient appointments and day stays, hospital admissions, and additional scans requested) and mode of delivery, will be costed using nationally published sources. The cost of the PlGF-based test under investigation will also be included for the women who consented to receive the revealed measurement. Descriptive statistics will be reported including mean cost per mother and infant, and 95% confidence intervals constructed using bootstrapping [25]. Mean cost and resource use per mother/infant dyad will also be reported by the PlGF-based test result. Missing data will be handled in the same way as the other statistical analyses.

### End of trial

The end of the trial will be defined as the date when the trial database is locked. An end-of-trial declaration will be made to the approving research ethics committee.

### Patient confidentiality, data handling, and record keeping

Overall responsibility for ensuring that each participant’s information is kept confidential will lie with the study sponsor. All paper documents will be stored securely and kept in strict confidence in compliance with the Data Protection Act (2018) and the General Data Protection Regulation. Data entered onto the electronic case report forms will be automatically transferred for storage in an electronic database held by MedSciNet on behalf of the sponsors. This information will be collected and retained with the participant’s explicit consent to enable the participant to be followed through the trial.

Due to the nature of pregnancy research, data will be kept for a period of no fewer than 25 years to allow follow-ups on health-related issues that may become relevant. All personal data will always be held securely and will not be used for any other purpose.

The dataset will be available to appropriate academic parties on request from the chief investigator in accordance with the data-sharing policies of King’s College London, with input from the co-investigator group where applicable.

### Quality control and assurance

#### Site initiation and training

The site PI and local research midwife or nurse, or their delegates, from each recruiting centre will be fully trained in the protocol and data collection procedures. They will then be responsible for delivering this training to all relevant site staff to make sure they understand the trial’s procedures prior to opening that site for recruitment. The site research team will also promote the trial and encourage recruitment so that the necessary recruitment targets are reached.

#### Site monitoring and auditing

The site research team will be responsible for the day-to-day smooth running of the trial at a recruiting site. The central trial team will monitor recruitment against targets, provide education and training, and monitor the completeness and quality of the data collected. The central trial team will visit recruiting centres and verify source data for a minimum of 10% of participants.

Throughout the trial, there will be central monitoring, overseen by the Project Management Group, Data Monitoring Committee, and Trial Steering Committee to ensure there is good communication between the central trial team and site staff. The DMC will look regularly at protocol adherence by site and by trial arm, including randomisation processes and patterns of allocation.

## Discussion

Current guidance in the UK at the time of trial commencement for management of suspected preterm pre-eclampsia recommends a single PlGF-based test at first presentation. There is high-quality evidence from randomised controlled trials that this improves management and reduces severe maternal adverse outcomes. Interventions that improve perinatal adverse outcomes in preterm pre-eclampsia are lacking. The role of repeat PlGF-based testing is uncertain and there is only preliminary evidence investigating this. This was a specific recommendation in the National Institute for Health and Care Excellence diagnostic guidance [12] and remains an active research question. This primary objective of this trial is to investigate whether repeat PlGF-based testing decreases a composite of perinatal severe adverse outcomes, and the results are likely to influence clinical practice in the management of suspected preterm pre-eclampsia.

## Trial status

The current PARROT-2 protocol is version 3.0 (26 January 2021). The trial received approval from REC/HRA on 1 November 2019. The trial opened to recruitment on 5 December 2019, and the first participant was recruited on 17 December 2019. Recruitment is ongoing and we are intending to complete recruitment by 30 September 2022.

## Supplementary Information

**Additional file 1.** Participant information leaflet

**Additional file 2.** Consent form

**Additional file 3.** Management algorithm

**Additional file 4.** Statistical analysis plan

**Additional file 5.** Health economics analysis plan

## Data Availability

The primary responsibility for preparing publications will lie with the chief investigators, Professor Lucy Chappell and Dr Louise Webster. All publications using data from this trial for original analyses will be submitted to the Trial Steering Committee for review before release. The research will be published in high-impact, peer-reviewed, scientific journals. More general dissemination of the results will be achieved through publication of summary findings. There are no commercial or intellectual rights issues that would delay the publication of results. A writing committee drawn from the co-investigators, trial coordinators, and others substantially involved in execution, analysis, and interpretation will be named authors on the principal publications arising from the trial, provided they meet the authorship criteria used by most high-impact peer-reviewed journals (see http://www.icmje.org). No external professional writers will be used. Local principal investigators will be named formally as collaborators on the publication; other trial personnel with significant input to the running of the trial will be named in the Acknowledgements in publications. The chief investigators will nominate and agree appropriate authorship on all publications prior to commencement of writing. Participants will be sent a summary of trial publications if they wish, with a reference to the final paper. A copy of the journal article will be made available to them on request from the chief investigators. Information will be made available on the trial website, including the final report and any publications when available. To target the clinical community, the results of this research will be disseminated at conventional academic platforms, including presentations at prominent national and international conferences. Requests for the final dataset can be made through the chief investigators in accordance with the data-sharing policies of King’s College London, with input from the co-investigator group where applicable.

## Abbreviations

PlGF: Placental growth factor
sFlt-1: Soluble fms-like tyrosine kinase-1
CI: Confidence interval
DMC: Data Monitoring Committee
TSC: Trial Steering Committee
SAE: Serious adverse event
ITU: Intensive care unit
PI: Principal investigator
Co-I: Co-investigator
PMG: Project Management Group
HRA: Health research authority
KCL: King’s College London
REC: Research ethics committee
NICE: National Institute for Health and Care Excellence
ISSHP: International Society for Study of Hypertension in Pregnancy
APEC: Action on Pre-eclampsia
