# Supplementary material for: Placental growth fActor Repeat sampling for Reduction of adverse perinatal Outcomes in women with suspecTed pre-eclampsia: study protocol for a randomised controlled trial (PARROT-2)
Source: Trials. 2022 Sep 2;23:722. doi: 10.1186/s13063-022-06652-8 (PMC9437393; doi:10.1186/s13063-022-06652-8)
Supplement: Supplementary file 1 — Additional file 1. Participant information leaflet [file 13063_2022_6652_MOESM1_ESM.pdf]

Placental growth fActor Repeat sampling for Reduction of adverse perinatal Outcomes in women with suspecTed pre-eclampsia: the PARROT-2 Trial

Participant Information Leaflet

You are being invited to take part in the study because you are between 22^{+0} and 35^{+6} weeks pregnant and your doctor or midwife thinks you might have pre-eclampsia.

This study, which fits into your normal clinical care, asks for you to give extra tubes of blood at the same time as your routine blood tests. Your doctor may use the result to guide your care. This study may improve the care of women with suspected pre-eclampsia in the future.

Pre-eclampsia is a common and potentially serious complication of pregnancy, which is thought to start in the placenta and can affect both the mother and her baby. The diagnosis is made from monitoring blood pressure and blood/urine tests at antenatal visits throughout the pregnancy. Early diagnosis of pre-eclampsia can reduce complications for the mother and her baby.

The first PARROT study has shown that changes in blood levels of a substance produced by the placenta called Placental Growth Factor (PlGF) can help reduce the time it takes for doctors and midwives diagnose pre-eclampsia. This test can also reduce harm to pregnant women who are diagnosed with pre-eclampsia. We do not know when or how often this test should be repeated to help continue monitoring pregnant women who are thought to be at increased risk of pre-eclampsia.

The PARROT-2 study will help doctors and midwives understand when and how often to repeat the PlGF-based blood test between 22^{+0} and 35^{+6} weeks and we hope this will improve the care of all women at risk of pre-eclampsia. 1280 women with pre-eclampsia from several hospitals in the UK will be involved in this study over 2 years. We will also analyse whether the repeat tests are good value in terms of cost or savings.

- **Our team will go through the information with you and answer any questions. This should take about 10-20 minutes and you can take as long as you need to decide if you want to take part.**
- **Joining the study is entirely up to you and you can stop taking part in the study at any time.**
- **This information leaflet explains what will happen if you choose to take part and is for you to keep.**
- **The care you receive from doctors and midwives will not be affected if you choose to take part in the study or not.**
- **Please discuss the study with friends and family if you would like to.**

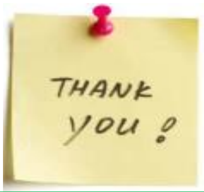

Thank you for taking the time to read this leaflet – please ask us if you would like more information

Tommy's
Funding research
Saving babies' lives

Do I have to take part? What will happen if I don't want to continue with the study?

No. It is entirely your decision whether to take part in the study. If you decide not to take part, your and your baby's care will not be affected in any way. **If you decide to take part you can change your mind at any time, without giving a reason.** We will ask if the information already collected can still be used.

If you decide not to take part in the trial of repeat testing for PlGF, we would like to ask your consent to follow up what happens to you and your baby.

What will happen if I do take part?

If you agree to take part, you will be asked to sign a consent form, and details about you and your pregnancy will be put into a secure computer database. This will also be noted in your hospital maternity records. The study computer will then select for you to have repeat PlGF-based blood tests with the results known or not known to you, your doctors and your midwives while you are pregnant. There will be a 50:50 chance of you being in either study group. Dividing people into groups like this is called a 'randomised clinical trial'. It is the standard and most reliable way of comparing different ways of caring for women in pregnancy. You will then have routine bloods to test for pre-eclampsia that will include your first PlGF-based test. Your doctor will then use the results of these tests to guide your care following their hospital standard practice.

Depending on the result of your blood tests (including your PlGF-based test), your doctors and midwives will decide if you need to be admitted to hospital or how often they will need to see you again in your pregnancy to make sure you and your baby are okay. When you are asked to have repeat blood tests for your routine follow up care, we will ask you for an extra 10mls or two teaspoons of blood for a PlGF-based test. Depending on which study group you are in, your doctors and midwives will be given or not given the result of the PlGF-based test. If your doctors and midwives are given the PlGF-based test result, they can use this to guide your care, in addition to following their hospital standard practice. If your doctors and midwives are not given the PlGF-based test results your care will be exactly the same as if you were not taking part in the study. You will only be asked to provide extra blood sample for this study once per week or once every two weeks (depending on the result of the first test) and only for a maximum of four times during your pregnancy.

When the study is finished, we will look at whether it was useful to your doctors and midwives to know your repeat PlGF-based test result. After you have given birth, you and your baby will be cared for in the usual way at your hospital. Information on your health will be collected about you and your baby until you are both discharged from hospital.

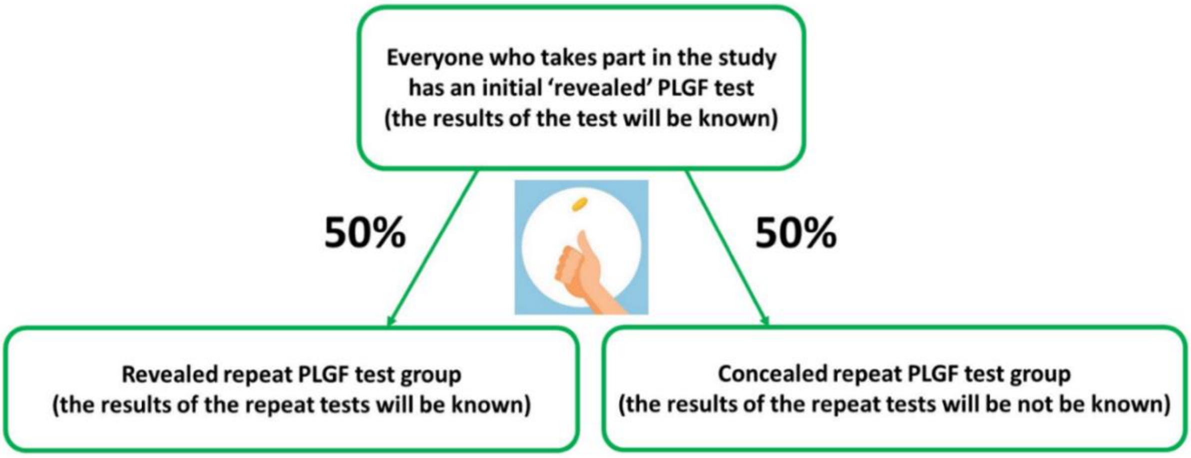

What are the possible risks and benefits of taking part?

There are no expected serious side effects to having the blood tests and you will be having blood tests as part of your normal clinical care. The first PARROT study showed that some women may spend less time in hospital; and if you have an abnormal result you may benefit from your doctors and midwives having more information about your pre-eclampsia condition.

Will taking part in this study be kept confidential?

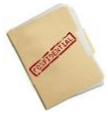

All the information collected about you and your baby during the study will be kept strictly confidential and is covered by the General Data Protection Regulation (GDPR). We will keep all information about you safe and secure. Electronic information is stored on a separate secure online database designed specifically for this study, and only accessible to those involved in the research, or checking the research is being done properly. You will not be directly identifiable from the study records but your initials will be included in your unique study code. To safeguard your rights, we will use the minimum personally-identifiable information possible. Unidentifiable data may be shared with researchers who are doing similar work, but it won't name you or your baby.

King's College London and Guy's & St Thomas' NHS Foundation Trust are the sponsors for this study and will act as the data controller. This means that we are responsible for looking after your information and using it properly. We will be using information from you and/or your or your baby's medical records in order to undertake this study. King's College London and Guy's & St Thomas' NHS Foundation Trust will keep identifiable information about you for 25 years after the study so that we can check the results of the trial. We will write our reports so that no-one can work out that you took part in the study.

Your rights to access, change or move your information are limited, as we need to manage your information in specific ways in order for the research to be reliable and accurate. If you withdraw from the study, we will keep the information about you that we already have.

With your permission, we will notify your GP of your involvement in this study.

You can find out more about how we use your information:

- By asking one of the research team
- By contacting the Data Protection Officer, Yinka Williams at DPO@gstt.nhs.uk
- At https://www.guysandstthomas.nhs.uk/research/patients/about.aspx
- At https://www.kcl.ac.uk/research/support/research-ethics/kings-college-london-statement-on-use-of-personal-data-in-research

What will happen to my blood sample after the tests for this study have been completed?

We would dispose of any remaining blood sample responsibly.

What if relevant new information becomes available?

Sometimes we get new information about the tests being studied. If this happens, any new information that is likely to affect your care or participation in the study will be discussed with you. You will be free to decide whether to continue with the study. If you decide to withdraw, your care and that of your baby will not be affected.

If the study is stopped for any reason, we will tell you and let you know what will happen next.

What if there is a problem?

If you have a concern about any aspect of this study, you should ask to speak to the researchers who will do their best to answer your questions [insert Principal Investigator name, telephone number and e-mail address]. If you remain unhappy and wish to complain formally, you can do this through the [add NHS site] Patients Advice and Liaison Service (PALS) on [add telephone number and email address]. The PALS team are based [add location for each NHS site].

In the event that something does go wrong and you suspect that the harm is the result of negligence by the Sponsor or Hospital, then you may be able to claim compensation from Guy's and St Thomas' NHS Foundation Trust and/or King's College London, but you may have to pay your legal costs. The normal National Health Service complaints mechanisms will still be available to you (if appropriate). King's College London also provides cover under its No Fault Compensation Insurance, which provides for payment of damages or compensation in respect of any claim made by a research participant for bodily injury arising out of participation in a clinical trial or healthy volunteer study (with certain restrictions).

What will happen to the results of the study?

The results and a full report will be published in a medical journal and on our website. If you like, you can be sent a short version of the final results. A copy of the full article can be requested from the Women's Health Division of King's College London. You or your baby will not be identified in any report or publication about the study.

Who is organising and funding the research?

The study is the responsibility of King's College London (KCL) and Guy's & St Thomas' NHS Foundation Trust and is being run by a research team in the Women's Health Division based at St Thomas' Hospital (details on the back page). The study is funded by Tommy's Baby Charity and the Moulton Charitable Trust. Your doctors and midwives will not be paid for enrolling you onto the study.

Have the public been involved in this study?

We asked members of Action on Pre-Eclampsia (APEC) and other Public Patient Involvement groups to help in the design of this study. We used feedback that they gave us in this leaflet.

Local contacts

Principal Investigator

{_LEAD_}

Local Research Midwife

{_MIDWIVES_}

Patient Advice and Liaison Services (PALS)

{_PALS_}

Chief Investigator: Professor Lucy Chappell

Women's Health Academic Centre
Division of Women's Health, 10th Floor North Wing,
St Thomas' Hospital, London SE1 7EH
T: 020 7188 9853 E: parrot@gstt.nhs.uk

REC Reference: 19/EE/0322 ISRCTN: 85912420

The PARROT study is funded by Tommy's and the Moulton Charitable Trust
