# Supplementary material for: Placental growth fActor Repeat sampling for Reduction of adverse perinatal Outcomes in women with suspecTed pre-eclampsia: study protocol for a randomised controlled trial (PARROT-2)
Source: Trials. 2022 Sep 2;23:722. doi: 10.1186/s13063-022-06652-8 (PMC9437393; doi:10.1186/s13063-022-06652-8)
Supplement: Supplementary file 2 — Additional file 2. Consent form [file 13063_2022_6652_MOESM2_ESM.pdf]

Consent Form

PARROT-2

Placental growth fActor Repeat sampling for Reduction of adverse perinatal Outcomes in women with suspecTed pre-eclampsia:

the PARROT-2 Trial

Hospital name: ______________________

Name of Principal Investigator: ______________________

Participant's Study Number: ______________________

Please
INITIAL BOX
DO NOT TICK

1. I confirm that I have read and understand the Participant Information Leaflet dated _ _/_ _/_ _ (Version _._) for this study and agree to take part. I have had the opportunity to consider the information and ask questions which have been answered satisfactorily. ☐
2. I understand that my participation in this study is voluntary and that I am free to withdraw from the study at any time without giving any reason, and without my medical care or legal rights, or that of my baby, being affected. ☐
3. I understand that relevant sections of my and my baby's medical records and data collected during the study may be looked at by the study organisers (members of the research team at King's College London), the research sponsors, or the NHS Trust. I give permission for these individuals to have access to these records where it is relevant to taking part in this research. ☐
4. I agree to blood samples being taken where it is relevant to taking part in this research. I understand that any part of these samples that are not used will be disposed of responsibly as per local laboratory guidelines. ☐
5. I agree that personal identifiable information will be collected, stored and used to follow my progress through the study and enable follow-up. This is on the understanding that all information will be treated confidentially. ☐
6. I understand that if I wish to have a copy of the lay report of this study, I will need to provide an email address that will be shared with King's college London. ☐

Name of Participant	Signature of Participant	D D / M M / Y Y
Name of Healthcare Professional taking consent	Signature of Healthcare Professional taking consent	D D / M M / Y Y

KING'S College LONDON

Women's Health Academic Centre,
Division of Women's Health,
10th Floor North Wing, London SE1 7EH
T: 020 71889853

Tommy's
Funding research
Saving babies' lives

Guy's and St Thomas' NHS
NHS Foundation Trust

The PARROT-2 study is funded by Tommy's Baby Charity and the Moulton Charitable Trust.

One copy to site file - one copy to participant's notes - one copy to participant
