# Supplementary material for: Placental growth fActor Repeat sampling for Reduction of adverse perinatal Outcomes in women with suspecTed pre-eclampsia: study protocol for a randomised controlled trial (PARROT-2)
Source: Trials. 2022 Sep 2;23:722. doi: 10.1186/s13063-022-06652-8 (PMC9437393; doi:10.1186/s13063-022-06652-8)
Supplement: Supplementary file 3 — Additional file 3. Management algorithm [file 13063_2022_6652_MOESM3_ESM.pdf]

PARROT-2

Hypertension in Pregnancy NICE Guidelines (June 2019)

with additional diagnostic test(s) for the PARROT-2 trial

Management of hypertension in pregnancy PRIOR to diagnosis of pre-eclampsia

**Maternal Monitoring**		
**BP**	**140/90–159/109 mmHg**	**≥160/110 mmHg**
Offer pharmacological treatment to all women with a BP above 140/90mmg. Aim for BP ≤135/85 mmHg		
**BP Monitoring**	1-2 per week until BP ≤135/85 mmHg	Every 15–30 mins until BP <160/110 mmHg
**Proteinuria Monitoring**	1-2 times per week	Daily
**Blood Tests**	FBC, U&E and LFT at presentation and then weekly<br>***PlGF based testing on one occasion when pre-eclampsia is suspected***	
**Admission to Hospital**	Do not admit routinely	Admit
**Assessment of Fetal Wellbeing**		
**Fetal Heart Auscultation**	Every appointment	Every appointment
**Ultrasound**	Repeat 2-4 weekly and at diagnosis of pre-eclampsia	Repeat 2 weekly and at diagnosis of pre-eclampsia
**CTG**	Only if clinically indicated	Only if clinically indicated and at diagnosis of pre-eclampsia

****INTERPRETATION OF PLGF RESULT****

****LOW OR VERY LOW PLGF RESULT IS NOT AN INDICATION FOR DELIVERY IN ITSELF****

	**PlGF ≥100 NORMAL**	**PlGF 12-99 LOW**	**PlGF <12 VERY LOW**
**Interpretation**	Test negative – normal. Pre-eclampsia ruled out. Highly unlikely to need delivery due to pre-eclampsia within 14 days.	Test positive – abnormal. Pre-eclampsia not ruled out. Increased risk for preterm delivery.	Test positive - highly abnormal. Assess as pre-eclampsia. Increased risk for preterm delivery.
**What does it mean?**	98% of women who are in this green range will not need delivery for pre-eclampsia within 14 days.	A PlGF test result <100 pg/ml will correctly identify 95-96% of women with pre-eclampsia who need delivery within 14 days.	Group at highest risk of preterm delivery and fetal growth restriction. 94% of women presenting before 35 weeks with PlGF <12 pg/ml will give birth preterm.
**Median time to delivery**	< 35 weeks: 62 days<br>35-37 weeks: 16 days	< 35 weeks: 23 days<br>35-37 weeks: 9 days	< 35 weeks: 9 days<br>35-37 weeks: 4 days
**Plan**	Continue with antenatal care as clinically indicated in combination with NICE guidance above	Consider increased surveillance, with regular monitoring and fetal ultrasound if indicated.	Assess as 'pre-eclampsia' (regardless of proteinuria) with increased surveillance and fetal ultrasound as indicated.

Additional Guidance for Management:
Hypertension in pregnancy: diagnosis and management https://www.nice.org.uk/guidance/ng133
Small-for-Gestational-Age Fetus, Investigation and Management https://www.rcog.org.uk/globalassets/documents/guidelines/gtg_31.pdf

Version 1.2 2020_02_14

Hypertension in Pregnancy NICE Guidelines (June 2019)

with additional diagnostic test(s) for the PARROT-2 trial

Management of hypertension in pregnancy PRIOR to diagnosis of pre-eclampsia		
**Maternal Monitoring**		
**BP**	**140/90–159/109 mmHg**	**≥160/110 mmHg**
Offer pharmacological treatment to all women with a BP above 140/90mmg. Aim for BP ≤135/85 mmHg		
**BP Monitoring**	1-2 per week until BP ≤135/85 mmHg	Every 15–30 mins until BP <160/110 mmHg
**Proteinuria Monitoring**	1-2 times per week	Daily
**Blood Tests**	FBC, U&E and LFT at presentation and then weekly<br>*sFlt-1/PlGF based testing on one occasion when pre-eclampsia is suspected*	
**Admission to Hospital**	Do not admit routinely	Admit
**Assessment of Fetal Wellbeing**		
**Fetal Heart Auscultation**	Every appointment	Every appointment
**Ultrasound**	Repeat 2-4 weekly and at diagnosis of pre-eclampsia	Repeat 2 weekly and at diagnosis of pre-eclampsia
**CTG**	Only if clinically indicated	Only if clinically indicated and at diagnosis of pre-eclampsia

****INTERPRETATION OF sFlt-1/PLGF RESULT****

****High sFlt-1/PLGF RESULT IS NOT AN INDICATION FOR DELIVERY IN ITSELF****

	**sFlt-1/PlGF ≤38 NORMAL**	**sFlt-1/PlGF 39-84 HIGH**	**sFlt-1/PlGF ≥ 85 VERY HIGH**
**Interpretation**	Test negative – normal. Pre-eclampsia ruled out. Highly unlikely to develop pre-eclampsia within 7 days.	Test positive – abnormal. Pre-eclampsia not ruled out. Increased risk for preterm delivery.	Test positive - highly abnormal. Assess as pre-eclampsia. Increased risk for preterm delivery
**What does it mean?**	99% of women who are in this green range will not develop pre-eclampsia within 7 days.	sFlt-1/PlGF test >38 will correctly identify 86% of women with pre-eclampsia who need delivery within 7 days.	The red range highlights those at highest risk of preterm delivery, including delivery of an SGA infant. SFlt-1/PlGF >85 correctly identifies 93% of women with pre-eclampsia who need delivery within 3 weeks.
**Median time to delivery**	51 days	17 days	
**Plan**	Continue with usual management	Consider increased surveillance, with regular monitoring and fetal ultrasound if indicated.	Assess as 'pre-eclampsia' (regardless of proteinuria) with admission for assessment, increased surveillance and fetal ultrasound if indicated.

Additional Guidance for Management:

Hypertension in pregnancy: diagnosis and management https://www.nice.org.uk/guidance/ng133

Small-for-Gestational-Age Fetus, Investigation and Management https://www.rcog.org.uk/globalassets/documents/guidelines/gtg_31.pdf
