# Supplementary material for: Placental growth fActor Repeat sampling for Reduction of adverse perinatal Outcomes in women with suspecTed pre-eclampsia: study protocol for a randomised controlled trial (PARROT-2)
Source: Trials. 2022 Sep 2;23:722. doi: 10.1186/s13063-022-06652-8 (PMC9437393; doi:10.1186/s13063-022-06652-8)
Supplement: Supplementary file 4 — Additional file 4. Statistical analysis plan [file 13063_2022_6652_MOESM4_ESM.docx]

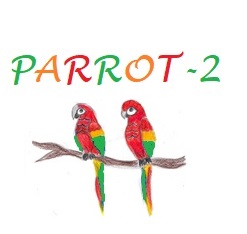


**Placental growth fActor Repeat sampling for Reduction of adverse perinatal Outcomes in women with suspecTed pre-eclampsia**

**The PARROT-2 Trial**

**Chief-Investigators: Professor Lucy Chappell**

**Dr Louise Webster**

ISRCTN: 85912420

REC: 19/EE/0322

**Statistical Analysis Plan**

**V1.0**

**Authors:** Dr Alice Hurrell (Trial Coordinator) and Mr Paul Seed (Trial Statistician)

**Reviewers:** Professor Lucy Chappell and Dr Louise Webster


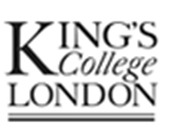

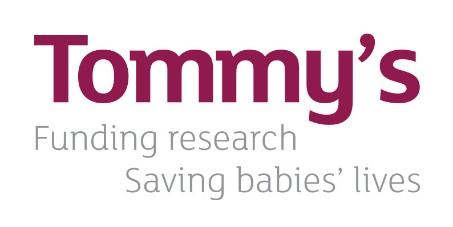

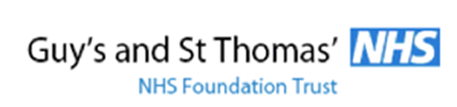


Contents

[2 Introduction 4](#_Toc66096515)

[3 Background 4](#_Toc66096516)

[3.1 Rationale 4](#_Toc66096517)

[3.2 Aims 5](#_Toc66096518)

[3.3 Trial design 6](#_Toc66096519)

[3.4 Eligibility 6](#_Toc66096520)

[3.4.1 Inclusion criteria 6](#_Toc66096521)

[3.4.2 Exclusion criteria 7](#_Toc66096522)

[3.5 Intervention 7](#_Toc66096523)

[4 Description of primary and secondary outcomes 7](#_Toc66096524)

[4.1 Primary outcome 7](#_Toc66096525)

[4.2 Secondary outcome 8](#_Toc66096526)

[5 Sample size and power 10](#_Toc66096527)

[6 Randomisation 11](#_Toc66096528)

[7 Protocol deviations 11](#_Toc66096529)

[7.1 Major 11](#_Toc66096530)

[7.2 Minor 11](#_Toc66096531)

[8 Participant groups for analysis 12](#_Toc66096532)

[8.1 Post-randomisation exclusions 12](#_Toc66096533)

[8.2 Descriptive analysis population 13](#_Toc66096534)

[8.3 Comparative analysis population 13](#_Toc66096535)

[8.4 Interim analysis population 13](#_Toc66096536)

[9 Descriptive analyses 14](#_Toc66096537)

[9.1 Representativeness of trial population and participant throughput 14](#_Toc66096538)

[9.2 Baseline comparability of randomised groups 14](#_Toc66096539)

[9.3 Loss to follow up 14](#_Toc66096540)

[10 Comparative analyses 14](#_Toc66096541)

[10.1 Primary outcome 15](#_Toc66096542)

[10.2 Secondary outcomes 15](#_Toc66096543)

[10.3 Health economic analysis 15](#_Toc66096544)

[10.4 Pre-specified subgroup analyses 16](#_Toc66096545)

[10.5 Significance levels 16](#_Toc66096546)

[10.6 Missing data 16](#_Toc66096547)

[10.7 Statistical software 16](#_Toc66096548)

[11 Safety data analysis 17](#_Toc66096549)

[12 Additional exploratory analysis 17](#_Toc66096550)

[13 References 18](#_Toc66096551)

# Introduction

This document details the proposed presentation and main analysis of the Tommy’s / J P Moulton Charitable Foundation funded multi-centre randomised controlled trial PARROT-2.

The results reported in the main paper(s) will follow the strategy set out here. Subsequent analyses of a more exploratory nature will not be bound by this strategy, although they will follow the broad principles laid down here. The principles are not intended to curtail exploratory analysis, nor to prohibit accepted practices, but they are intended to establish the rules that will be followed, as closely as possible, when analysing and reporting the trial.

The analysis plan will be available on request when the principal manuscripts are submitted for publication. Suggestions for subsequent analyses by journal editors or reviewers will be considered carefully and carried out, as far as possible, in line with the principles of this analysis plan.

Any deviations from the statistical analysis plan will be described and the rationale given in the final report of the trial. The analysis will be carried out by an identified, appropriately qualified and experienced statistician, who will ensure the integrity of the data during processing. Examples of such procedures include quality control and evaluation procedures.

# Background

## Rationale

Hypertension affects 10% of pregnant women, with pre-eclampsia affecting 2.8% of singleton pregnancies.(1, 2) Maternal complications include death, eclampsia, liver rupture, stroke, pulmonary oedema, and acute kidney injury, and fetal/neonatal complications include stillbirth, fetal growth restriction, and iatrogenic preterm delivery.(3) Pre-eclampsia has a variable clinical presentation but is likely to be more severe when it develops before 37 weeks’ gestation (preterm pre-eclampsia) and women may be asymptomatic even with severe disease. Symptoms may progress over weeks and do not correlate well with diagnosis or clinical outcomes. Conversely, hypertension and proteinuria can occur without progression to pre-eclampsia. Pre-eclampsia also doubles a woman’s lifelong risk of cardiovascular disease.

Placental growth factor (PlGF) is an angiogenic protein synthesized in the placenta. Concentration of PlGF in maternal blood rises across gestation to peak at 30 weeks and then falls slightly towards term in uncomplicated pregnancy. PlGF >5^th^ centile ($\geq$100 pg/ml) below 37 weeks’ gestation rules out pre-eclampsia that will require birth of the baby within 14 days.(4) PlGF-based testing has recently been recommended by the National Institute for Health and Care Excellence (NICE) for ruling out pre-eclampsia within 14 days.(1) Our group recently published the findings of the PARROT trial demonstrating that a single PlGF test in women on presentation with suspected preterm pre-eclampsia decreased time to diagnosis of pre-eclampsia (median 1.9 days with intervention versus 4.1 days with usual care; hazard ratio 0.39, 95%CI 0.17 to 0.91 by parametric survival analysis).(5) We additionally demonstrated a reduction in maternal severe adverse outcomes from 5.4% with usual care to 3.8% with the intervention (adjusted odds ratio 0.32; 95%CI 0.11 to 0.96), and reduced use of high-level neonatal care facilities when the test was integrated into clinical management.(5)

## Aims

The aim of the trial is to investigate repeat PlGF-based testing in women with suspected preterm pre-eclampsia.

The primary objective is:

- To establish whether repeat PlGF-based testing decreases a composite of perinatal severe adverse outcomes, in women who have already had a first PlGF-based test.

The secondary objectives are:

- To determine if repeat PlGF-based testing reduces secondary perinatal and maternal adverse outcomes.
- To assess the health resource use associated with repeat-PlGF based testing in a budget impact analysis.
- To establish the diagnostic test accuracy of abnormal ‘low’ (PlGF <100 pg/ml, further sub-divided into the ‘very low’ (PlGF <12 pg/ml) category), or abnormal ‘high’ sFlt-1/PlGF ratio (>38) repeat PlGF-based tests in predicting pre-eclampsia requiring delivery in 14 days.

## Trial design

The PARROT-2 trial is designed as a pragmatic, randomised, controlled, multi-centre trial of repeat revealed PlGF-based testing compared to usual care with repeat concealed testing, in women presenting with suspected preterm pre-eclampsia between 22^+0^ and 35^+6^ weeks of gestation inclusive, with a primary outcome of a composite of perinatal severe adverse outcomes. All women will have initial PlGF-based testing at presentation (as recommended by the National Institute for Health and Care Excellence(1)). Women will be randomised at an individual level. The trial will be conducted in approximately 15 to 30 consultant-led maternity units across England and Scotland.

Women who do not wish to participate in the trial will still be offered an initial revealed PlGF-based test as part of their investigations for suspected preterm pre-eclampsia (in line with guidance from the National Institute for Health and Care Excellence(1, 2)) and those accepting this test will be asked for written consent to collection of an observational outcome data set which will be used to assess generalisability of the trial findings. Analysis of this cohort is outside the remit of this Statistical Analysis Plan.

## Eligibility

### Inclusion criteria

Women will be considered eligible for inclusion into the trial if they fit the following criteria at the time of the first PlGF-based test:

- Clinical suspicion of pre-eclampsia
- Pregnancy of between 22^+0^- and 35+6-weeks’ gestation inclusive
- Singleton pregnancy
- Viable fetus
- Women aged 18 years or more at the time of presentation
- Able to give written informed consent

### Exclusion criteria

Women will be excluded from the trial if they have a confirmed diagnosis of preterm pre-eclampsia at the time of the first PlGF-based test.

## Intervention

PlGF immunoassays are NICE-approved diagnostic tests for the initial assessment of suspected pre-eclampsia.(1, 2) All regulatory approvals are in place. PlGF and sFlt-1 are stable markers, and the collection of blood samples is straightforward, requiring no additional processes beyond centrifugation (as used in routine clinical blood sampling). Coefficients of variation have been established for the assay and are acceptable for use in clinical practice.

The results of the repeat PlGF-based test will be known to the health care professionals and the women in the revealed arm and used in addition to the other clinical features to inform ongoing management plan integrated with the National Institute for Health and Care Excellence Hypertension in Pregnancy Guideline.(1) Clinical staff will be trained in the interpretation of PlGF-based test results and provided with a management algorithm to integrate the result into the participants’ clinical care.

Each centre uses either the Roche or the Quidel version of the PlGF-based test (based on local clinical preference).

# Description of primary and secondary outcomes

## Primary outcome

The primary outcome is a composite of stillbirth (fetal death ≥24 weeks’ gestation), early neonatal death (within first 7 days of birth), or neonatal unit admission.

## Secondary outcome

Tested perinatal outcomes:

- Stillbirth
- Early neonatal death
- Neonatal unit admission
- Gestational age at delivery
- <10th birthweight centile (using Intergrowth-21^st^ standards)
- Survival to discharge without severe morbidity: defined as survival to neonatal discharge without any of the following: bronchopulmonary dysplasia, retinopathy of prematurity, severe necrotising enterocolitis, brain injury, late-onset sepsis (to be tested if the pooled event rate is at least 4% of the total, similar to the event rate reported in PARROT-1)

Additional descriptive perinatal outcomes:

- Late neonatal death (within 28 days of delivery)
- <3^rd^ Birthweight centile (using Intergrowth-21^st^ standards)
- Severe necrotising enterocolitis
- Sepsis (including early-onset sepsis and late-onset sepsis)
- Brain injury
- Seizures
- Retinopathy of prematurity
- Chronic lung disease or bronchopulmonary dysplasia
- Umbilical arterial pH at birth (where measured)

Maternal tested secondary outcomes (between enrolment and delivery):

- Severe adverse maternal outcome composite (defined by fullPIERS consensus)(6)
- Proportion of women diagnosed with pre-eclampsia (defined by International Society for Study of Hypertension in Pregnancy)(7)
- Systolic blood pressure ≥160 mmHg on at least one occasion post study enrolment and discharge
- Delivery mode (vaginal, assisted vaginal, caesarean section)
- Concealed first repeat PlGF-based test performance (with comparison against currently utilised tests) for clinically indicated delivery for pre-eclampsia within 14 days (including test performance stratified by first test result)

Additional descriptive maternal outcomes:

- Components of the fullPIERS composite:(6)
  - Maternal death
  - Eclampsia
  - Glasgow coma score <13
  - Stroke or reversible ischaemic neurological deficit
  - Transient ischaemic attack
  - Cortical blindness or retinal detachment
  - Posterior reversible encephalopathy
  - Positive inotropic support
  - Severe uncontrolled hypertension with parenteral infusion of third-line antihypertensive required
  - Myocardial infarction/ischaemia
  - Blood oxygen saturation <90%
  - Requirement of ≥50% FiO2 for >1 hour
  - Intubation required (other than for caesarean section)
  - Pulmonary oedema
  - Transfusion of blood products required
  - Platelet count <50 × 10⁹ platelets/L
  - Hepatic dysfunction
  - Hepatic haematoma or rupture
  - Severe acute kidney injury (creatinine >150 μmol/L; no pre-existing renal disease or creatinine >200 μmol/L; pre-existing renal disease))
  - Dialysis required
  - Placental abruption
- Abnormal fetal ultrasound features post enrolment:
  - estimated fetal weight <10th centile
  - oligohydramnios
  - absent or reversed umbilical artery Doppler end diastolic flow
- Labour onset (spontaneous, induced or pre-labour caesarean section)
- Indications for delivery
- Postpartum haemorrhage
- Repeat PlGF-based test results (number of repeat tests per woman, proportion changing category)

Health resource use outcomes for budget impact analysis:

- Maternal:
  - Antenatal outpatient attendances
  - Inpatient days
  - Intensive care unit days
- Perinatal:
  - Intensive care unit days
  - High dependency unit days
  - Special care unit days

The cost of repeat PlGF-based testing will be included for those in the intervention group.

# Sample size and power

Sample size was calculated using data from the PELICAN study and PARROT trial combined, (4, 5) demonstrating that 25.7% had the primary outcome (stillbirth, early neonatal death or neonatal unit admission). A sample size of 1208 women (604 participants per group) would have 90% power, at the 5% significance level, to detect an overall reduction of 30% (to 18%) in the composite primary outcome score. Although loss to follow-up in the PARROT-1 trial was three of 1023 women, we will allow for up to 5% loss to follow-up and plan to recruit 1268 women in this trial. If 3% of women are lost to follow-up, a sample size of 1244 would be sufficient.

This analysis will primarily assess a PlGF-based testing strategy, using one of two NICE-approved tests (Roche and Quidel).(2) If we recruit at least 650 women into the trial using each of the Roche PlGF-based test (sflt-1/PlGF), or the Quidel test, then if analysed as its own group, this would give 90% power to detect a reduction in the composite events from 25.7% to 15.4% (40% relative risk reduction) or 80% power to detect a reduction in the composite events from 25.7% to 16.7% (35% relative risk reduction).

Whilst we initially aimed to recruit 1208 women to one of the two PlGF-based testing strategies, the COVID-19 pandemic has impeded recruitment and therefore, we will primarily assess PlGF-based testing as a whole, with further analysis as outlined.

# Randomisation

Randomisation will be managed via MedSciNet, a secure web-based randomisation facility. The allocation ratio of intervention (repeat revealed PlGF-based testing) to control (repeat concealed PlGF-based testing) will be 1:1. Participants will usually be randomised as soon as they have signed consent to participate in the study. A minimisation algorithm will be used to ensure balance between the groups with respect to the maternity unit, gestational age at randomisation (22^+0^ to 27^+6^, 28^+0^ to 31^+6^, >32^+0^ weeks’ gestation), primary indication for testing (hypertension, other).

The MedSciNet web-based randomisation, using a minimisation algorithm as described above, will ensure that the allocation mechanism is concealed from researchers, clinicians, and participants. Allocation concealment will be ensured as the randomisation will not be released until the woman has been recruited into the trial. The randomisation algorithm will be checked prior to trial initiation.

Due to the study design, it is not possible to mask allocation from the clinical researchers or the women who are recruited to the trial. However, the study team will take steps to ensure that those participants assigned to the concealed repeat sampling arm of the trial do not have any repeat revealed tests.

# Protocol deviations

All protocol non-compliances will be summarised by trial arm in the final report.

## Major

The following will be defined as major protocol non-compliances:

- Data considered fraudulent

## Minor

The following will be defined as minor protocol non-compliances:

Participants randomised in error:

These include participants who were ineligible for the trial:

- Who are < 22^+0^ or ≥ 35^+6^ weeks' gestation on day of first PlGF-based test
- With a known lethal fetal anomaly
- With twins or higher-order multi-fetal pregnancy
- Aged under 18 years
- Whose consent to take part has not been documented
- Where a diagnosis of pre-eclampsia was established prior to first PlGF-based test
- Where the clinical eligibility criteria are not met

Participants who do not receive the intervention as planned:

These include participants:

- In whom a repeat blood sample was not obtained
- In whom a blood sample was obtained but a result was not processed or voided by the sample meter
- In whom a repeat blood sample was revealed in the concealed arm of the trial

# Participant groups for analysis

For all outcomes, the primary inference will be based on a pragmatic intention to treat (ITT) analysis, i.e., women will be analysed in the groups into which they were randomly allocated, regardless of allocation received. A secondary Per-Protocol (PP) analysis of the primary endpoint and the severe adverse maternal outcome composite will be restricted to women managed as randomised.

## Post-randomisation exclusions

Exclusions to the analysis population post randomisation consist of the following: -

- Women identified as non-eligible post-randomisation
- Women for whom a consent form was not received
- Women for whom an entire record of fraudulent data was detected

(Should fraudulent data be detected, consideration will be given to excluding all data for the site where such data was found).

Ineligible women randomised in error will be removed from the study. For the main analysis, women will be analysed in their allocated groups according to the Intention To Treat (ITT) principle.

The numbers (with percentages of the randomised population) of post-randomisation exclusions will be reported by randomised treatment group, and reasons summarised.

If a woman withdraws from the study, the data up until the point of withdrawal will be included (as specified in the patient information leaflet) and data following that time point will be reported as ‘missing’.

## Descriptive analysis population

Baseline demographic and clinical characteristics will be reported for all women randomised excluding post-randomisation exclusions (see section 8.1)

## Comparative analysis population

Perinatal outcomes

All infants born to a trial participant, by trial arm, excluding post-randomisation exclusions

Maternal outcomes

All women recruited, by trial arm, excluding post-randomisation exclusions.

## Interim analysis population

Different denominators will be used in the interim analysis:

- Baseline data will be reported for all trial participants with available data, excluding known post-randomisation exclusions.
- Outcome data will be reported for all women and babies of women who have delivered and been discharged home at the time of the database snapshot, excluding known post-randomisation exclusions.
- Safety data will be reported for all trial participants with available data, excluding known post-randomisation exclusions.

# Descriptive analyses

## Representativeness of trial population and participant throughput

The flow of participants through each stage of the trial will be summarised using a CONSORT diagram.(8) We will report the numbers of participants:

- Allocated to each intervention,
- Received allocated intervention,
- Withdrew consent to use data,
- Included in the analysis,
- Excluded from analysis,

## Baseline comparability of randomised groups

Women in the two randomised groups will be described separately with respect to maternal characteristics at trial entry. The number and percentage will be presented for binary and categorical variables. The mean and standard deviation or the median and the interquartile range will be presented for continuous variables, or the range if appropriate. There will be no tests of statistical significance performed nor confidence intervals calculated for differences between randomised groups on any baseline variable.

## Loss to follow up

The number and percentage of losses to follow up among women will be reported for the two trial arms, and the reasons will be recorded. Any deaths of the woman or baby and their causes will be reported separately.

# Comparative analyses

The main analysis will follow the intention to treat principle and will use all available information on participants who accepted randomisation, irrespective of later protocol deviations.

## Primary outcome

The binary composite primary outcome of stillbirth, early neonatal death, or neonatal unit admission will be analysed using binomial regression with a log link, adjusted for the minimisation variables (maternity unit, gestational age at randomisation (22^+0^ to 27^+6^, 28^+0^ to 31^+6^, >32^+0^ weeks’ gestation) and primary indication for testing (hypertension, other). Results will be presented as a risk ratio with 95% confidence intervals. Logistic regression and odds ratios will only be used if the binomial model fails to converge.

## Secondary outcomes

The tested secondary perinatal and maternal outcomes (as defined in section 4.2) will be analysed using log binomial regression models (as section 10.1) and results will be presented as adjusted risk ratios with 95% confidence intervals. Continuous outcomes will be analysed using linear regression with log transformations as necessary. Additional perinatal and maternal outcomes will be reported using descriptive statistics alone.

Within the concealed group, the diagnostic accuracy of the first repeat sample will be assessed for pre-eclampsia requiring delivery in 14 days (the commonly used outcome in previous diagnostic test accuracy studies). Sensitivity, specificity, positive and negative predictive value, positive and negative likelihood ratios, using cut points of 12 and 100 pg/mL, and area under the receiver operating characteristic curve, will be reported with 95% confidence intervals.

Longitudinal gestational profiles of repeated measures PlGF and sFlt-1 and sFlt-1/PlGF will be plotted in relation to normal ranges. Appropriate receipt of antenatal corticosteroids will be described.

## Health economic **analysis**

A full health economic analysis plan will be written prior to locking the dataset.

In brief, data on mother and infant, antenatal and post-natal acute hospital care (hospital attendances including outpatient appointments and day stays, hospital admissions, surgery and diagnostic tests including additional scans requested) and mode of delivery, will be costed using nationally published sources. The cost of the PlGF-based test under investigation will also be included for the women consented to receive the revealed measurement. Descriptive statistics will be reported including mean cost per mother and infant, and 95% confidence intervals constructed using bootstrapping using by the Bias correction and acceleration method (BCa).(9)

Mean cost and resource use per mother/infant dyad will also be reported by PlGF-based test result. Missing data will be handled in the same way as the other statistical analyses.

## Pre-specified subgroup analyses

Planned subgroup analyses will be carried out to check whether the intervention effect differs between groups identifiable at baseline. These will include gestation at time of first test (≤35 vs >35 weeks’ gestation), first PlGF result (<100 and ≥100 pg/mL), first sFlt-1:PlGF ratio (≤38 or >38) and primary indication for testing (hypertension, other).

The subgroup analyses will be limited to the primary perinatal outcome and the severe adverse maternal outcome composite. Interaction tests will be carried out to check whether there is a significant difference between the subgroups.

## Significance levels

For all analyses, a 95% confidence interval will be calculated.

## Missing data

Missing data will be described, for example, by presenting the number and percentage of individuals in the missing category. Based on previous experience, we will treat missing data as missing at random (MAR) and with adjustment for the minimisation variables.

## Statistical software

The statistical software Stata (StataCorp, College Station, Texas, USA), version 16 or later will be used for all analyses.

# Safety data analysis

Serious Adverse Events will be summarised by trial arm, with numbers of each severity (mild, moderate or severe); relatedness (unrelated, possibly, probably, definitely); and action taken (to be categorised when data is available).

# Additional exploratory analysis

Any analyses not specified in the analysis protocol will be exploratory in nature and a two-sided significance level of 0.01 will be used with 99% confidence intervals. All such analyses will be approved by the Co-investigator Group.

# References

1. NICE. Hypertension in Pregnancy: diagnosis and management. <https://www.nice.org.uk/guidance/ng1332019>.

2. NICE. PlGF-based testing to help diagnose suspected pre-eclampsia (Triage PlGF test, Elecysys immunoassay sFlt-1/PlGF ratio, DELFIA Xpress PlGF 1-2-3 test, and BRAHMS sFlt-1

Kryptor/BRAHMS PlGF plus Kryptor PE ratio). <https://www.nice.org.uk/guidance/dg232016>.

3. Mol BW, Roberts CT, Thangaratinam S, Magee LA, De Groot CJ, Hofmeyr GJ. Pre-eclampsia. The Lancet. 2016;387(10022):999-1011.

4. Chappell LC, Duckworth S, Seed PT, Griffin M, Myers J, Mackillop L, et al. Diagnostic Accuracy of Placental Growth Factor in Women With Suspected PreeclampsiaClinical Perspective. Circulation. 2013;128(19):2121-31.

5. Duhig KE, Myers J, Seed PT, Sparkes J, Lowe J, Hunter RM, et al. Placental growth factor testing to assess women with suspected pre-eclampsia: a multicentre, pragmatic, stepped-wedge cluster-randomised controlled trial. Lancet. 2019;393(10183):1807-18.

6. von Dadelszen P, Payne B, Li J, Ansermino JM, Broughton Pipkin F, Côté AM, et al. Prediction of adverse maternal outcomes in pre-eclampsia: development and validation of the fullPIERS model. Lancet. 2011;377(9761):219-27.

7. Brown MA, Magee LA, Kenny LC, Karumanchi SA, McCarthy FP, Saito S, et al. Hypertensive Disorders of Pregnancy: ISSHP Classification, Diagnosis, and Management Recommendations for International Practice. Hypertension. 2018;72(1):24-43.

8. Schulz KF, Altman DG, Moher D. CONSORT 2010 statement: updated guidelines for reporting parallel group randomised trials. BMC medicine. 2010;8(1):18.

9. Efron B, Halloran E, Holmes S. Bootstrap confidence levels for phylogenetic trees. Proc Natl Acad Sci U S A. 1996;93(23):13429-34.
