# Supplementary material for: Placental growth fActor Repeat sampling for Reduction of adverse perinatal Outcomes in women with suspecTed pre-eclampsia: study protocol for a randomised controlled trial (PARROT-2)
Source: Trials. 2022 Sep 2;23:722. doi: 10.1186/s13063-022-06652-8 (PMC9437393; doi:10.1186/s13063-022-06652-8)
Supplement: Supplementary file 5 — Additional file 5. Health economics analysis plan [file 13063_2022_6652_MOESM5_ESM.docx]

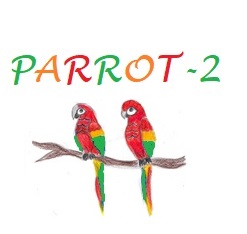


**Placental growth fActor Repeat sampling for Reduction of adverse perinatal Outcomes in women with suspecTed pre-eclampsia**

**The PARROT-2 Trial**

**Chief-Investigators: Professor Lucy Chappell**

**Dr Louise Webster**

ISRCTN: 85912420

REC: 19/EE/0322

Health Economics Analysis Plan

**Version 0.1**

**27 April 2022**

Protocol Version 3.0, 26^th^ January 2021

SAP version 1.0, 17^th^ April 2021

Author: Rachael Hunter (Associate Professor of Health Economics, University College London)

Reviewer: Professor Lucy Chappell (Chief Investigator)

### Contents

###

[1. Introduction 2](#_Toc101968852)

[2. Background and Trial Design 3](#_Toc101968853)

[3. Intervention 4](#_Toc101968854)

[4. Aim 4](#_Toc101968855)

[5. Outcomes 4](#_Toc101968856)

[6. Resource use and costing 5](#_Toc101968857)

[7. Procedure for accounting for missing data 5](#_Toc101968858)

[8. Discounting 5](#_Toc101968859)

[9. Analysis 5](#_Toc101968860)

[9.1 Budget Impact Analysis 5](#_Toc101968861)

[9.2 Cost-effectiveness analysis 5](#_Toc101968862)

[9.3 Cost-effectiveness plane and cost-effectiveness acceptability curve 6](#_Toc101968863)

[10. Sensitivity Analyses 6](#_Toc101968864)

[11. Subgroup analysis 6](#_Toc101968865)

[12. References 6](#_Toc101968866)

[13. Approvals 8](#_Toc101968867)

[14. Document history 8](#_Toc101968868)

# Introduction

This document details the health economic analysis for the trial PARROT-2, a multicentre randomised trial funded by Tommy’s / J P Moulton Charitable Foundation. The results reported in this publication will follow the strategy set out here. Subsequent analyses of a more exploratory nature will not be bound by this strategy, although they are expected to follow the broad principles described.

The health economics analysis plan will be available on request when the principal manuscripts are submitted for publication. Suggestions for subsequent analyses by journal editors or referees will be considered carefully and carried out, as far as possible, in line with the principles of this analysis plan. The statistical analysis will be described in a separate analysis plan.

Any deviations from the health economics analysis plan will be described and the rationale given in the final report of the trial. The analysis will be carried out by an identified, appropriately qualified and experienced health economist, who will ensure the integrity of the data during processing.

**Chief and Principal Investigator**

Professor Lucy Chappell and Dr Louise Webster

Women’s Health Academic Centre, King’s College London

[lucy.chappell@kcl.ac.uk](mailto:lucy.chappell@kcl.ac.uk) and louise.m.webster@kcl.ac.uk

**Trial Statistician**

Paul Seed

Women’s Health Academic Centre, King’s College London

[paul.seed@kcl.ac.uk](mailto:paul.seed@kcl.ac.uk)

**Trial Coordinator**

Alice Hurrell

Women’s Health Academic Centre, King’s College London

[alice.hurrell@kcl.ac.uk](mailto:alice.hurrell@kcl.ac.uk)

**Health Economist**

Rachael Hunter

Health Economics Analysis and Research methods Team (HEART), University College London

[r.hunter@ucl.ac.uk](mailto:r.hunter@ucl.ac.uk)

# Background and Trial Design

PARROT-2 is a pragmatic, multi-centre, randomised, controlled trial to investigate repeat PlGF-based testing in women with suspected preterm pre-eclampsia between 22^+0^ and 35^+6^ weeks of gestation, inclusive. The primary objective is to establish whether repeat PlGF-based testing decreases a composite of perinatal severe adverse outcomes, in women who have already had a first PlGF-based test.

All women will have initial PlGF-based testing at presentation (as recommended by the National Institute for Health and Care Excellence (1)), with women randomised at an individual level. The trial will be conducted in approximately 20 to 30 consultant-led maternity units across England and Scotland. The trial aims to recruit 1268 participants.

Women who do not wish to participate in the trial will still be offered an initial revealed PlGF-based test as part of their investigations for suspected preterm pre-eclampsia (in line with guidance from the National Institute for Health and Care Excellence (1, 2)) and those accepting this test will be asked for written consent to collection of an observational outcome data set which will be used to assess generalisability of the trial findings. Analysis of this cohort is outside the remit of the Statistical and Health Economics Analysis Plans.

An economic evaluation using Monte-Carlo analysis and data from PARROT, a cluster randomised stepped wedge trial of PlGF testing and a clinical management algorithm, found that clinical care with PlGF testing cost £149 less than standard care. The majority of the cost-savings were due to a reduction in outpatient appointments for women testing with a PlGF>100pg/ml (women presenting as high risk for pre-eclampsia but with a “rule out” result). The results though are potentially dependent on the cost per test.

# Intervention

PlGF immunoassays are NICE-approved diagnostic tests for the initial assessment of suspected pre-eclampsia. (1, 2) All regulatory approvals are in place. PlGF and sFlt-1 are stable markers, and the collection of blood samples is straightforward, requiring no additional processes beyond centrifugation (as used in routine clinical blood sampling). Coefficients of variation have been established for the assay and are acceptable for use in clinical practice.

The results of the repeat PlGF-based test will be known to the health care professionals and the women in the revealed arm and used in addition to the other clinical features to inform ongoing management plan integrated with the National Institute for Health and Care Excellence Hypertension in Pregnancy Guideline.(1) Clinical staff will be trained in the interpretation of PlGF-based test results and provided with a management algorithm to integrate the result into the participants’ clinical care.

Each centre uses either the Roche sFlt-1/PlGF or the Quidel PlGF version of the PlGF-based test (based on local clinical preference).

# Aim

The primary aim of the health economic analysis is to estimate the mean incremental cost of repeat PlGF and ongoing management of suspected preterm pre-eclampsia between 22^+0^ and 35^+6^ weeks of gestation compared to a single PlGF test only (concealed arm). The analysis uses patient level trial data collected from clinical records as part of PARROT-2 and is from the maternity service cost perspective. The time horizon of the analysis is from initial PlGF-based test until maternal and infant discharge following delivery or death.

The health economic analysis will follow the statistical analysis plan (SAP v1.0 April 2021) in retaining the validity of the randomisation process and Protocol version 3 (26/01/2021). The primary analysis will be intention-to-treat (ITT) where all randomised patients are analysed in their allocated group whether or not they received their allocated treatment. Sample size calculations, exclusion criteria, randomisation procedures, definitions of protocol non-compliance, analysis of the primary clinical outcome and descriptive statistics or demographic variables are set out in the SAP.

# Outcomes

The following outcomes collected from clinical records will be used to calculate costs for the economic evaluation:

Maternal: antenatal outpatient attendances and inpatient days; intensive care unit use

Perinatal: intensive care, high dependency and special care unit days

# Resource use and costing

Descriptive statistics will be reported for the percentage of mothers/infants and mean number of contacts for each type of resource use by randomised arm. Resource use will be costed based on unit costs obtained from the National Schedule of NHS Costs. (3)

The cost of the repeat PlGF test will be based on information provided by the test providers (Roche and Quidel) and will only be applied to participants randomised to repeat revealed PlGF only. The initial PlGF test will not be costed as it is the same in both arms. The primary analysis will calculate the cost of the PlGF test based on the actual test utilised. As part of the sensitivity analysis (see section 10) the cost of the PlGF test will be varied for a range of values to see what impact it has on the results.

# Procedure for accounting for missing data

It is assumed that women and infants missing resource use items did not receive that resource use. As data comes from routine medical records the amount of missing data is likely to be low and missing at random. The analysis will be complete case.

# Discounting

As the time horizon of the analysis is from recruitment until maternal and perinatal discharge or death and hence less than 12-months no discounting is required.

# Analysis

Linear regression will be used to calculate the adjusted difference in resource use and costs between arms for each resource use category and for maternal and infant costs adjusting for test provider and gestation at randomisation.

95% confidence intervals will be calculated from bias corrected and accelerated bootstrap results.

## Budget Impact Analysis

The primary analysis will calculate the mean incremental cost per woman/infant dyad of revealed repeat PlGF test compared to no repeat PlGF test (concealed arm). 95% confidence intervals will be calculated from bias corrected and accelerated bootstrap results, adjusting for test provider and gestation at randomisation. Costs will include all maternal and infant costs for all randomised participants in line with restrictions set out in with this analysis plan and the SAP as well as the cost of repeat PlGF in the revealed arm.

## Cost-effectiveness analysis

The mean incremental cost per gain in primary outcome, as defined by the SAP, will be calculated if revealed repeat PlGF costs more and is more effective than no repeat PlGF test based on a statistically significant result for the primary outcome. If repeat PlGF costs less and is significantly more effective it will be reported as dominant. For all other instances only costs will be reported, with a note on the primary outcome result.

## Cost-effectiveness plane and cost-effectiveness acceptability curve

The bootstrapped data from budget impact analysis (9.1) and the primary outcome will be used to construct a cost-effectiveness plane and cost-effectiveness acceptability curve if the requirements set out in 9.2 are met.

# Sensitivity Analyses

The mean incremental cost as described in 9.1 will be calculated for a range of costs per repeated PlGF test.

# Subgroup analysis

Costs differences between revealed and concealed PlGF will be reported for the following subgroups: gestation at time of first test (≤35 vs >35 weeks’ gestation), gestation at delivery (≤35 vs >35 weeks’ gestation), first PlGF result (<100 and ≥100 pg/mL), first sFlt-1:PlGF ratio (≤38 or > 38) and indication for testing (hypertension or other).

An analysis by index of multiple deprivation (IMD) quintiles will also be conducted.

# References

1. NICE. Hypertension in Pregnancy: diagnosis and management. <https://www.nice.org.uk/guidance/ng1332019>.

2. NICE. PlGF-based testing to help diagnose suspected pre-eclampsia (Triage PlGF test, Elecysys immunoassay sFlt-1/PlGF ratio, DELFIA Xpress PlGF 1-2-3 test, and BRAHMS sFlt-1

Kryptor/BRAHMS PlGF plus Kryptor PE ratio). <https://www.nice.org.uk/guidance/dg232016>.

3. NHS England. NHS National Schedule of NHS costs 2019-2020. <https://www.england.nhs.uk/costing-in-the-nhs/national-cost-collection/>

# Approvals

| **Chief Investigator** | | | |
| --- | --- | --- | --- |
| **Print Name:** | Professor Lucy Chappell | **Signature:** |  |
| **Affiliation:** | Women’s Health Academic Centre,  St Thomas’ Hospital | **Date:** |  |
| **Health Economist** | | | |
| **Print Name:** | Rachael Hunter | **Signature:** |  |
| **Affiliation:** | Primary Care and Population Health, University College London | **Date:** |  |

# Document history

| Version | Date | Edited by | Comments |
| --- | --- | --- | --- |
| 0.1 | 27/04/2020 | RH | First draft. |
